# Exposure to 3G mobile phone signals does not affect the biological features of brain tumor cells

**DOI:** 10.1186/s12889-015-1996-7

**Published:** 2015-08-08

**Authors:** Yu-xiao Liu, Guo-qing Li, Xiang-ping Fu, Jing-hui Xue, Shou-ping Ji, Zhi-wen Zhang, Yi Zhang, An-ming Li

**Affiliations:** Department of Neurosurgery, First Affiliated Hospital of PLA General Hospital, 51 Fushi Road, Beijing, People’s Republic of China; China Telecommunication Technology Labs, Beijing, China; Department of Cell Biology, Institute of Basic Medical Sciences, 27 Taiping Road, Beijing, 100850 People’s Republic of China; Department of blood molecular biology, Institute of blood transfusion medicine, Beijing, China

**Keywords:** Electromagnetic fields, Cell phone, Glioblastoma cells, Growth, Apoptosis, Migration

## Abstract

**Background:**

The increase in mobile phone use has generated concerns about possible risks to human health, especially the development of brain tumors. Whether tumor patients should continue to use mobile telephones has remained unclear because of a paucity of information. Herein, we investigated whether electromagnetic fields from mobile phones could alter the biological features of human tumor cells and act as a tumor-promoting agent.

**Methods:**

Human glioblastoma cell lines, U251-MG and U87-MG, were exposed to 1950-MHz time division-synchronous code division multiple access (TD-SCDMA) at a specific absorption rate (maximum SAR = 5.0 W/kg) for 12, 24, and 48 h. Cell morphologies and ultra-structures were observed by microscopy and the rates of apoptosis and cell cycle progression were monitored by flow cytometry. Additionally, cell growth was determined using the CKK-8 assay, and the expression levels of tumor and apoptosis-related genes and proteins were analyzed by real-time PCR and western blotting, respectively. Tumor formation and invasiveness were measured using a tumorigenicity assay *in vivo* and migration assays *in vitro*.

**Results:**

No significant differences in either biological features or tumor formation ability were observed between unexposed and exposed glioblastoma cells. Our data showed that exposure to 1950-MHz TD-SCDMA electromagnetic fields for up to 48 h did not act as a cytotoxic or tumor-promoting agent to affect the proliferation or gene expression profile of glioblastoma cells.

**Conclusions:**

Our findings implied that exposing brain tumor cells *in vitro* for up to 48 h to 1950-MHz continuous TD-SCDMA electromagnetic fields did not elicit a general cell stress response.

**Electronic supplementary material:**

The online version of this article (doi:10.1186/s12889-015-1996-7) contains supplementary material, which is available to authorized users.

## Background

With the increasing use of mobile phones worldwide, electromagnetic fields (EMF) exposure from mobile phones has become a serious potential public health problem. The risk of developing intracranial tumors induced by mobile phone EMF exposure is of particular interest. Although it is agreed that tumorigenesis is linked to physical stress and environmental alterations, the nature or existence of carcinogenic effects related to EMF exposure have remained unclear [1–5].

To date, most epidemiological studies have not demonstrated an increased risk of brain tumor development with overall mobile phone usage [6–9]. However, some positive associations have been reported, such as a relationship between an increased risk of acoustic neuroma and long-term mobile phone use [10, 11]. To date, recent data have shown a controversial effect of high frequency EMF on the biological features of tumor cells *in vitro* [12–14]. This conclusion was based on the lack of a solid biological mechanism, and the fact that brain cancer rates are not significantly increasing [15]. Notably, it remains uncertain whether mobile phone exposure is linked to the development of brain tumors. Furthermore, there is little evidence available about the effects of mobile phone use on the progression of disease in tumor patients.

Previously, we investigated the effects of 1950-MHz time division-synchronous code division multiple access (TD-SCDMA) exposure on the growth of normal rat glia cells and found that continuous exposure to a 1950-MHz TD-SCDMA EMF might damage normal astrocytes [16]. Therefore, we wanted to further study the relationship between mobile phone use and the risk of human glioblastoma development. The defining criteria for known neuron-carcinogenic agents include the following: (a) a capability to increase the growth rate of tumor cells or inhibit apoptosis; (b) a capability to increase the invasiveness of tumor cells; and (c) a capability to promote the formation of human tumor cells *in vivo* [17]. This present study was designed to determine whether TD-SCDMA, a type of 3G technology that is widely employed in China at a specific absorption rate (SAR), could elicit an effect on principal cellular processes in a neural tumor system. The sensitivities of different glioblastoma-derived cell lines, including T98G, A127, U251-MG, and U87-MG cells, to 1950-MHz TD-SCDMA EMF exposure were examined using cell growth and apoptosis assays. Then, U251-MG and U87-MG cells were used to further study the biological effects of TD-SCDMA EMF exposure *in vitro* and *in vivo*. The first aim of this study was to evaluate whether 1950-MHz TD-SCDMA exposure affected the common biological features of glioblastoma cells *in vitro*, including cell proliferation, apoptosis, morphology, ultra-structure, cell cycle, and migration. The second objective was to confirm the gene expression profiles of the human glioblastoma cell lines, U251-MG and U87-MG, which were exposed to TD-SCDMA EMF. The final objective was to investigate whether EMF from mobile phones altered the tumor formation ability of these cells *in vivo*.

## Methods

### Cell culture

The human glioblastoma cell lines U251-MG, U87-MG, T98G,A127 were obtained from the Biological Cell Institute of Chinese Peking Union Medical College (Chinese Academy of Medical Science, Beijing). Before use, approval was given by the institutional ethics committee of Chinese PLA General Hospital (Beijing, China). Cell were cultured in 25 cm RPMI 1640 medium containing 10 % fetal calf serum (Qianzhaoxinye Life Science & Technology Inc, Beijing) 100 U/mL penicillin and 100 g/mL streptomycin at 37 °C and 5 % CO_2._ Cells of different groups were plated into 25-mm dish at 2 × 10^5^ cells/dish 24 h before exposure.

### Exposure system

**Fig. 1:**
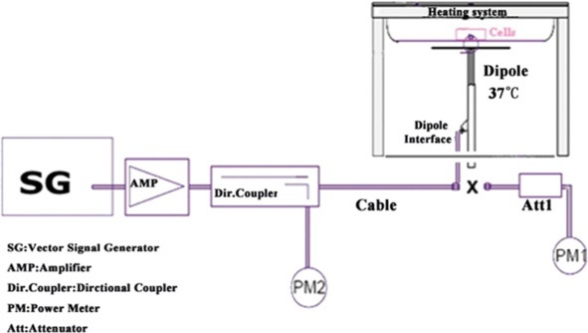
Exposure system setup. The vector signal generator generates a modulated TD-SCDMA signal. The amplifier is used to amplify the original signal. The power meter (PM2) attached to a directional coupler can provide an additional monitor path for stable exposure. First, the power meter PM1 (including the attenuator, Att1) is connected to the cable to measure the forward power at the location of the connector (x) to the dipole. The signal generator is adjusted to the desired forward power at the connector, as read by power meter PM1, after attenuation (Att1), which also was coupled to PM2. After connecting the cable to the dipole, the signal generator was readjusted for the same reading at power meter PM2 as before. Throughout the exposure period, the reading of PM2 was kept constant to ensure that the power feed into the dipole was stable, which guaranteed stable exposure

The exposure system was provided by China Telecommunication Technology Labs as shown in Fig. 1. The experimental cells (up to four bottles simultaneously) cultured in 25-cm cell culture bottles containing L-15 medium (medium independent of CO2, Gibco™, Invitrogen Corporation) were subjected to the microwave exposure generated from dipole antenna (SPEAG D1900V3-SN1118, Swiss Federal Institute of Technology (ETH), Zurich, Switzerland) fixed under the cell plates. The antennas were connected to a vector signal source plus a power amplifier, which emitted a 1950-MHz radiofrequency (RF) electromagnetic field at the same frequency as TD-SCDMA mobile phones,i.e., a 1950-MHz carrier frequency, which is the median value of TD-SCDMA mobile phones of Band A (2010–2025 MHz) and Band F (1880–1920 MHz). The power fed to the antenna was 250 mW (the nominal maximum power). The parameters were qualified by a forward power sensor and meter before the experiment, and monitored by a backward power sensor and meter during the whole exposure experiment to keep the drift within The specific absorption rate, corresponding to the energetic flux absorbed by the cell culture, was 5.0 W/kg, which is the maximum value averaged over 10 g cell culture. The method used to determine the SAR value is numerical simulation calculation on HFSS. The cell culture electrical parameters are measured 10 times at 37 °C and the average values are*σ* = 2.14 Ω/m *ε*_*r*_ = 71.8 and *ρ* = 1 000 g/m^3^. These values are used in the numerical simulation calculation. Simulation model is as Additional file 1: Figure S3A. In simulation, the input power is 250 mW, and the frequency is 1950 MHz. In HFSS software, the electrical strength components *Ex*, *Ey* and *Ez* can be got by solving the 3D FDTD equations, and then get the grid points’ SAR by formula:

$$ SAR=\frac{\sigma {E_i}^2}{\rho } $$

Because 10 g cell culture includes 2400 grid points, 10 g averaged SAR can be got then.

The calculation data are as Additional file 1: Figure S3B. Simulation data show that the SAR distribution mode in the cell culture is that when the distance to the container bottom varies from 0 mm to 72 mm, the 10 g averaged SAR non-uniformly decreases from the maximum value 5.0 W/kg to almost 0.

As comparison, when this simulation method is used in standard SAR measurement model, which represents the SAR calibration scenario by SPEAG, the calculation results are: SARpeak = 12.8 W/kg, SAR(10 g) = 4.8 W/kg. Compared with SPEAG calibration results, i.e., SARpeak = 12.2 W/kg, SAR(10 g) = 5.0 W/kg, provide by the certificate, the error are 4 % and 6.5 % respectively, which validate the simulation method.

As RF exposure is required to be conducted under 37 °C, an appropriate heating system was used to keep steady the temperature of the cell plates at 37 °C. Background of magnetic fields in incubators is an importance factor in cell culture work [18]. Here, the maximum background of the magnetic field in the incubator was 0.7μT. In each experiment, cells were divided into 4 groups: unexposed group, exposed for 12, 24 and 48 h. At the end of the exposure, the samples were simultaneously removed and processed for cell biological tests, total mRNA, whole cell and nuclear protein extractions (Fig. 1).

### Observation of cell morphology and ultra-structure

The morphology of cells in different groups was observed by microscopy. In addition, the ultrastructure of cells was observed using transmission electron microscopy (TEM). Briefly, cells were fixed for 4 h at 4 °C in 5 % glutaraldehyde, washed three times in 0.1 mol/L phosphate buffered saline (PBS), post-fixed for 2 h at 4 °C in 2 % osmium tetroxide, dehydrated in a graded series of ethanols and embedded in Epon 812. Tissue was cut into ultrathin sections (75 nm) and then stained with uranyl acetate and lead citrate. Sections were viewed with a HITACHI H-600 electron microscope at 80 kV (HITACHI, Tokyo, Japan).

### Staurosporine treatment

Cells were cultured in 6-well culture plates until 80 % confluence was reached and then they were treated for 24 h with 100 nM Staurosporine (Sigma) to induce apoptosis.

### Cell apoptosis assays

After exposure, both floating and adherent cells were collected and assessed of apoptosis. Briefly, the cells of different groups were washed twice with PBS and collected by trypsinization. After centrifugation at 1200rmp for 5 min at room temperature, the cells were stained with Annexin V and propidium iodide (1 mg/mL). The cell apoptosis distributions were determined on a FACScort flow cytometer and analyzed by ModFitLT V3.0 software program.

### Cell proliferation assay

After exposure, cells of different groups were seeded in 96-well plates at 5 × 10^3^cells/well. After that, cell proliferation was evaluated using the CCK-8 assay (C0038, Beyotime Inst Biotech, China) according to manufacturer’s instructions. Briefly, 10 μl of CCK8 solution was added to the culture medium, and incubated for additional 3 h. The absorbance was determined at 450 nm wave-length.

### Cell growth cycle

When cells grew to 50–60 % confluence, 2 mmol/L thymidine was added to the medium. After 11–16 h of culture, the culture medium was replaced with fresh medium. And the cells were exposed for 12, 24 and 48 h respectively. After exposure, cells in different groups were trypsinized and fixed with 70 % cold alcohol overnight at -20 °C, then the cells were washed with PBS and maintained in the presence of RNase-A enzyme for 30 min in a water bath at 37 °C. The cells were subsequently stained with propidium iodide (PI; 25ug/ml) for 30 min without light. Finally, the distribution of cells in the cycle was analyzed with a flow cytometer.

### Wound healing and invasion assays

After exposure, cell invasion was assessed by using Matrigel Invasion Chambers (BD Biosciences) and Wound-healing assays, as previously described [19]. Briefly,after exposure, cells were plated in trans-well chambers pre-coated with Matrigel Invasion Chamber medium, and containing 5 % fetal bovine serum (FBS) in the lower chamber as the chemo-attractant. After incubation for 24 h at 37 °C in a humidified incubator with 5%CO2, the non-invading cells were removed with cotton swabs. The invasive cells that attached to the lower surface of the membrane insert were fixed in 100%methanol at room temperature for 2 min and stained with DAPI (Invitrogen). The number of migrated cells on the lower surface of the membrane was calculated in microscopic fields. Images were acquired using a microscope.

Wound-healing assays were performed on cells after exposure. Cells were trypsinized and plated into 6-well plates at high densities. When the cells had reached 90 % confluency, a wound was made through the cells with a micropipette tip and photographs taken immediately (time zero) and 24 h after wounding for respectively. The distance migrated by the cell monolayer to close the wounded area during this time period was measured by ImageJ software. Results were expressed as a migration index-that is, the distance migrated by treated cells relative to the distance migrated by untreated cells. Experiments were carried out in triplicate and repeated at least five times.

### Real-time PCR

The gene expression of cells in different groups was examined with Real-Time PCR using the SYBR Green PCR Master Mix as previously described [20]. All PCR reactions were performed as follows: 95 °C for 5 min; 94 °C for 40 s; annealing at various temperatures for 40 s, 72 °C for 40 s (25 cycles); 72 °C for 10 min, 4 °C for 5 min. The forward and reverse primers used were described in Table 1.

**Table 1:** The primer sequences for Real-Time PCR

name	Primer sequence (sense)	Primer sequence (antisense)
*bcl-2*	tgcggattgacatttctg	atcccatcaatcttcagcac
*c-myc*	ctgcgacgaggaggagaa	ccgaagggagaagggtgt
*emp-1*	tcattgccctcctggtct	ttcaacggcttcctcctc
*bax*	gacggcaacttcaactggg	cctggatgaaaccctgaagc
*actin*	agagctacgagctgcctgac	agcactgtgttggcgtacag

### Western blotting analysis

Total proteins extracts of each group cells were resolved by 10 % SDS-PAGE and transferred on PVDF (Millipore) membranes. After blocking, the PVDF membranes were washed 4 times for 15 min with TBST at room temperature and incubated with primary antibody (rabbit anti-Id-1 polyclonal antibody Abcam). Following extensive washing, membranes were incubated with secondary peroxidase-linked goat anti-rabbit IgG (Santa Cruz) for 1 h. After washing 4 times for 15 min with TBST at room temperature once more, the immuno-reactivity was visualized by enhanced chemiluminescence (ECL kit, Pierce Biotechnology), and membranes were exposed to KodakXAR-5 films (Sigma-Aldrich).

### Tumorigenicity assays in nude mice

After exposure, a total of 1 × 10^6^ cells in different groups suspended in PBS were injected into male nude mice subcutaneously. Eight groups of mice (*n* = 3) were tested. Mice were sacrificed at 28 days post-injection. The quantity of tumors were excised and measured.

### Histology and immuno-histochemistry

Tumors from different groups were removed and fixed with formaldehyde, embedded in paraffin wax, and sectioned. Sections were incubated with mouse anti-Ki67 monoclonal antibodies (Santa Cruz) at 4 °C overnight, and then incubated with anti-mouse IgG for 1 h. Images were acquired using a microscope. Ki67-positive cells/in a field were calculated in 10 high power microscopic fields.

### Statistical analysis

Results were expressed as means ± SD. Statistical significance was determined by using Student t- test. Results were considered significant at *P* < 0.05.

## Results

### Effects of RF emission on apoptosis and proliferation of different glioblastoma cell lines

**Fig. 2:**
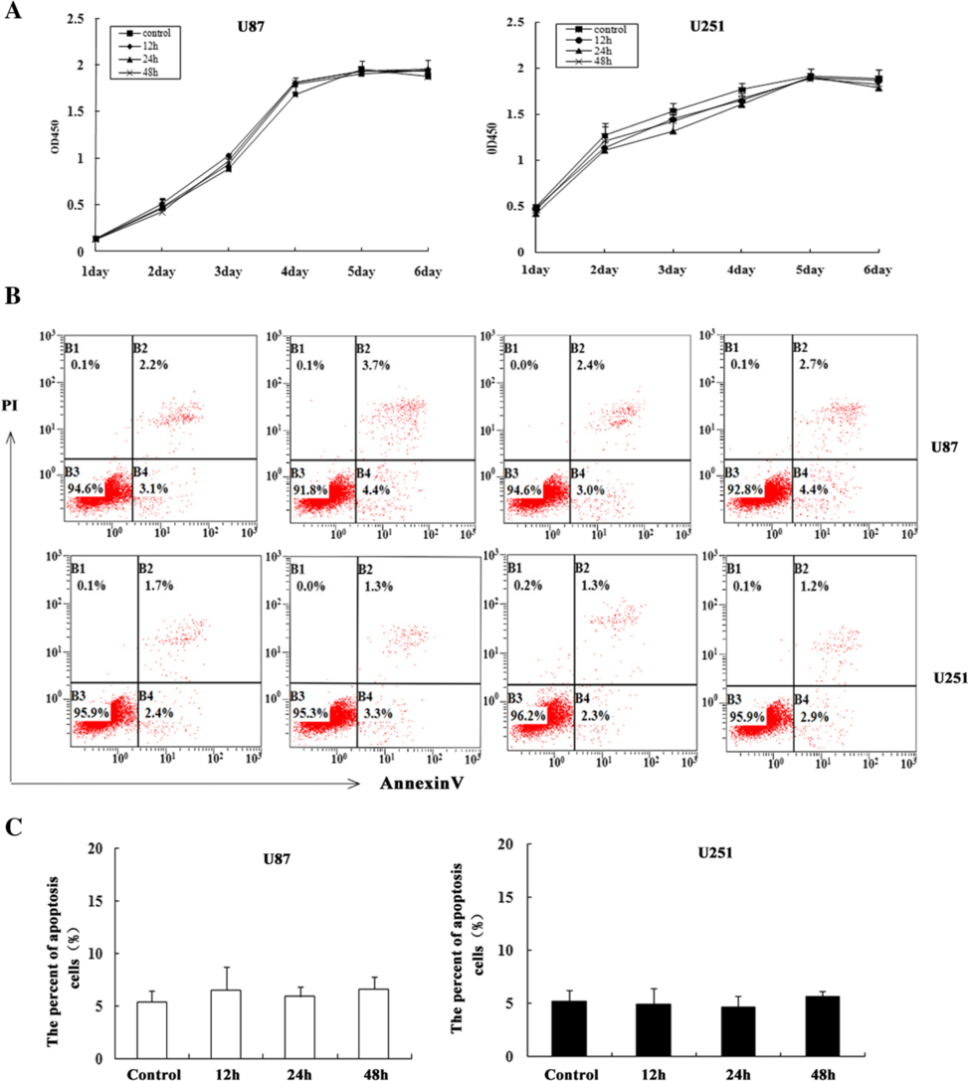
The growth and apoptosis of human glioma cells measured after exposure. **a** The proliferation of U87-MG and U251-MG cells was measured using the CKK-8 assay from days 1 to 6 after exposure. Data represent means ± SD from five independent experiments. **b** Apoptosis of U251-MG and U87-MG cells were detected using the FITC-conjugated Annexin-V/PI assay after exposure for 12, 24, or 48 h. **c** Statistical analysis of the percentage of apoptotic cells in the different groups. Data represent means ± SD from three independent experiments

First, we examined the sensitivity of different glioblastoma cell lines to1950-MHz TD-SCDMA EMF using cell apoptosis and proliferation assays. T98G, A127, U251-MG, and U87-MG cells were continuously exposed to 1950-MHz TD-SCDMA EMF for 48 h. Early and late apoptosis of cells in the different groups was determined using the Annexin V/propidium iodide assay at different time points(Additional file 2: Figure S1, Fig. 2b,c). For all glioblastoma cell lines we tested, there was no significant differences in early and late apoptosis were observed between the control and exposure groups(Additional file 2: Figure S1, Additional file 3: Table S1).

T98G, A127, U251-MG, and U87-MG cells grown in the logarithmic phase to ~60 % confluency were exposed to 1950-MHz TD-SCDMA EMF for 12, 24, and 48 h. Cells from the different groups were plated (5 × 10^3^ cells/well) in 96-well plates and cell viability was determined using the CKK-8 assay from days 1 to 6. As shown in Fig. 2a and Additional file 2: Figure S1A, the viability of human glioblastoma cells remained unchanged after continuous exposure for up to 48 h. A-127 and T98G cells were not found to be tumorigenic in *nude* mice. Thus, U251-MG and U87-MG were used in the subsequent more detailed studies.

### Effects of RF emission on the morphology and ultra-structure of glioblastoma cells

**Fig. 3:**
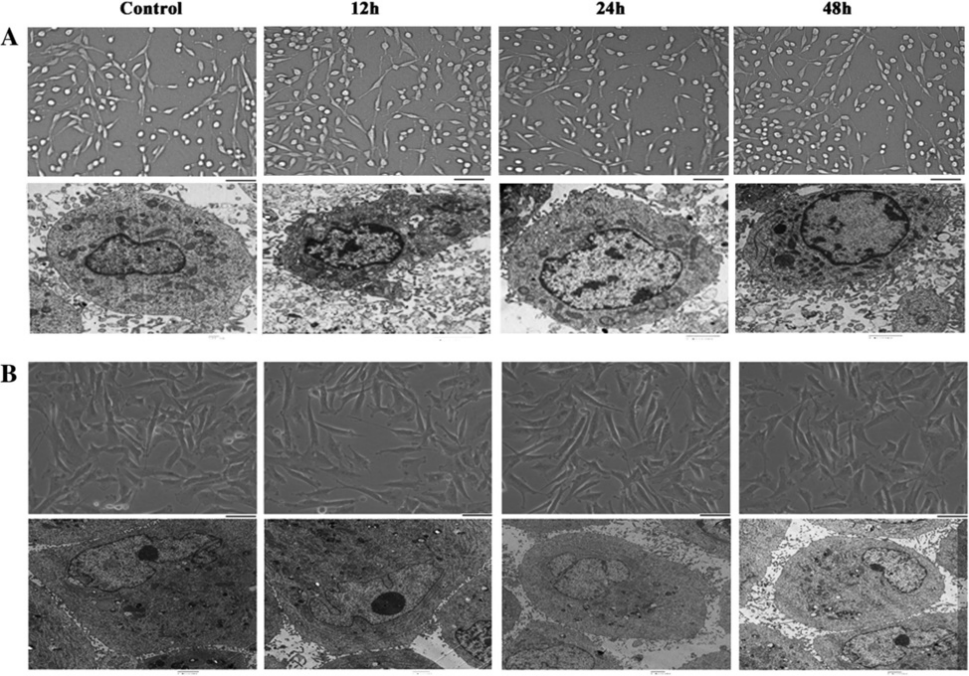
Effects of RF emission on the morphology and ultra-structure of human glioma cells. The morphology and ultra-structure of U251-MG (**a**) and U87-MG (**b**) cells were recorded after exposure for 12, 24, or 48 h. There were no significant differences between the control and treated groups. Scale bar, 100 μm

The human glioblastoma U251-MG and U87-MG cell lines were exposed to 1950-MHz TD-SCDMA EMF for 12, 24, or 48 h. After exposure, the morphology of the glioblastoma cells in different groups was observed by microscopy. Unexposed U251-MG cells were small, shuttle process-bearing cells with obvious synapses. The unexposed U87-MG cells had a similar appearance, but were larger. After exposure for 12, 24, or 48 h, the morphology of both cells did not appear to be different compared with the unexposed groups (Fig. 3).

The ultra-structure of cells in different groups was observed by transmission electron microscopy. Cells in the unexposed group had well-distributed nuclear chromatin, clear pericaryon, normal mitochondria, regular smooth endoplasmic reticulum, and rough endoplasmic reticulum without degranulation. There were no significant differences in the morphology of cells between the control and exposed groups, which was in accord with the morphology of the cells. These findings indicated that continuous exposure for up to 48 h of a 1950-MHz TD-SCDMA EMF may not induce structural changes in human glioblastoma cells (Fig. 3).

### Effects of RF emissions on the cell cycle of human glioblastoma cells

**Fig. 4:**
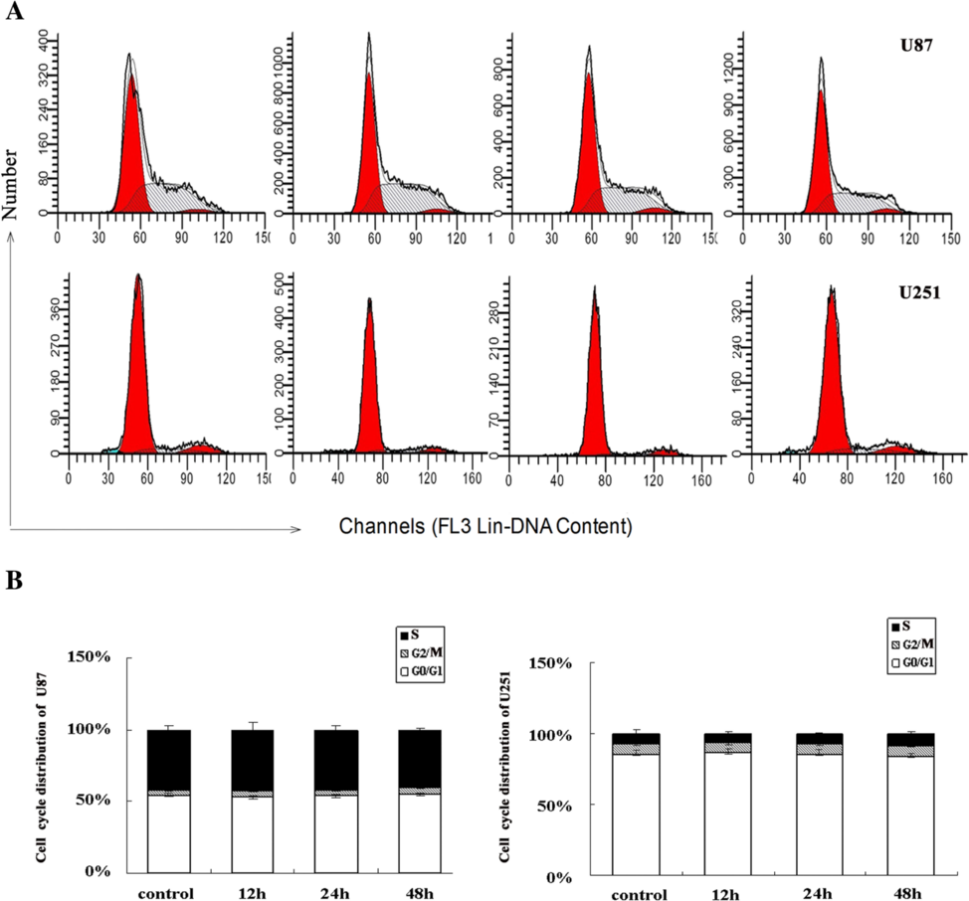
Cell cycle distribution of human glioma cells after exposure. **a** The proportion of cells in each cell cycle stage was measured by flow cytometry after exposure for 12, 24, or 48 h. **b** Statistical analysis of the proportions of cells in each cell cycle stage after exposure. Data represent means ± SD from five independent experiments

Then, the effects of RF exposure on cell cycle progression were examined (Fig. 4). For these experiments, U251-MG and U87-MG cells were incubated either in the absence or presence of RF exposure for 12, 24, and 48 h. At the end of each exposure, cells in the 12 and 24 h groups wecultured in an RF-free environment until 48 h post-exposure. The proportion of cells in the G0/G1, S, and G2/M phases was determined by flow cytometry using propidium iodide (PI). For U251-MG cells, ~80 % of cells in the unexposed group were observed to be in the G0-G1 phase after incubation for 48 h. No differences were observed in cell cycle distribution between the control and exposure groups e(Additional file 3: Table S2). For U87-MG cells, the proportion of cells in the G0-G1 phase was ~50 % and EMF exposure for 12, 24, or 48 h did not cause remarkable differences in the cell cycle(Additional file 3: Table S2).

### Effects of RF emissions on cell migration and invasion

**Fig. 5:**
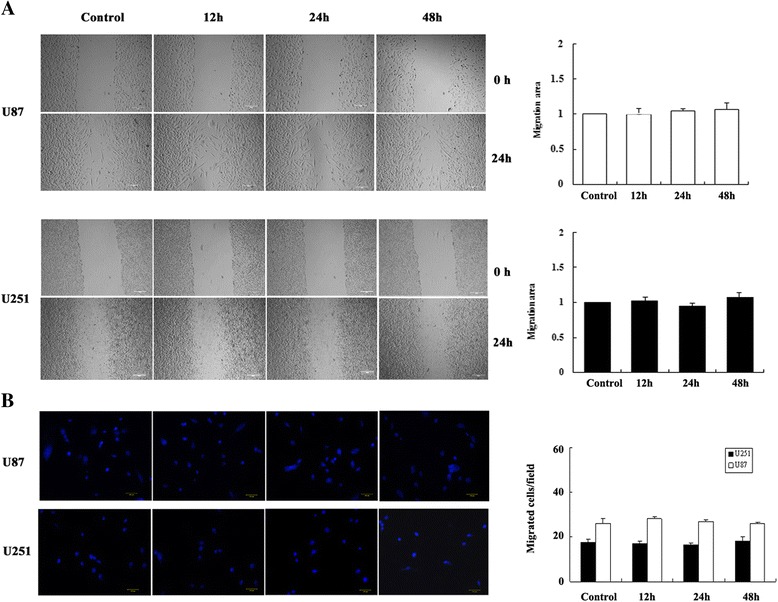
Observation of cell migration after exposure. Wound healing (**a**) and trans-well (**b**) assays were used to study cell migration. Migrated cells were quantified by capturing an average of 10 random fields from three independent duplicate experiments; the distance migrated by the cell monolayer to close the wounded area during this time period was measured. The migration index of each treatment, expressed as a value that related the distance traveled by treated cells to the distance migrated by untreated cells

Tumor migration and invasion ability are important standards for evaluating the malignancy levels of tumor cells. After exposure to EMF, 5 × 10^4^ U87-MG or U251-MG cells were plated in the upper compartment of trans-well chambers. After 24 h, cells were fixed and the numbers of cells in the lower chamber were counted. The migration rate of both U87-MG and U251-MG cells was not affected by RF exposure (Fig. 5b).

Wound healing assays were conducted to further estimate the invasion capacity of U87-MG and U251-MG cells after EMF exposure. We found that there was no significant difference in the wound healing capacity of U87-MG and U251-MG cells between the control and exposure groups (Fig. 5a). These data suggested that continuous exposure to 1950-MHz TD-SCDMA EMF for no more than 48 h might not alter the migration or invasion capacity of human glioblastoma cells.

### Effects of RF emissions on human glial tumor development and progression

**Fig. 6:**
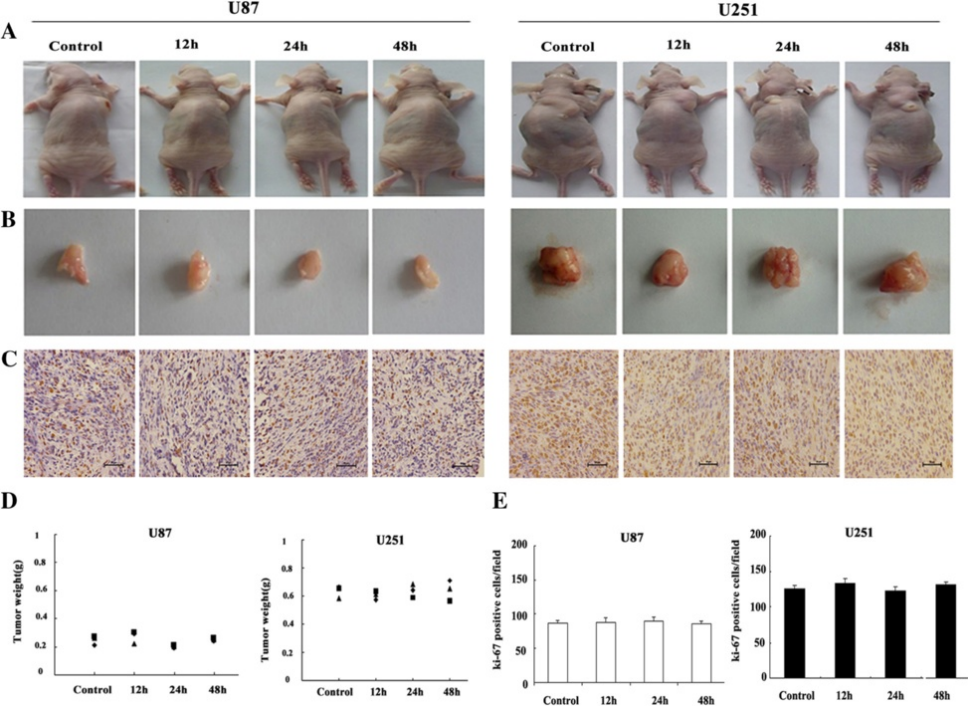
Tumorigenicity assays for human glioma cells. Tumorigenicity assays in *nude* mice (**a–b**). Tumor formation could be detected in all groups. **c** The expression of Ki-67 in tumors of different groups was measured by immunostaining. **d** Tumor weights in different groups. **e** Ki-67-positive cells were quantified by capturing an average of 10 random fields in the slices. Data represent means ± SD from three independent experiments

To investigate whether cell line-induced tumor formation might be affected by EMF exposure, 24 *nude* mice were inoculated with U251-MG or U87-MG cells from the control or experimental groups, and tumor size was monitored within 4 weeks after injection. At 10 days, tumors appeared in *nude* mice of all groups and tumor volumes increased during a 4-week follow-up period (Fig. 6a–b). The average relative tumor weight was measured for each group (Fig. 6d). We found that for both types of human glioblastoma cells, there was a significant difference in the average size and weight of tumors between the control and experimental groups after 12, 24, and 48 h exposure.

To investigate the effects of RF emissions on the proliferation of U87-MG and U251-MG cells *in vivo,* protein expression levels of the proliferation marker Ki-67 were measured using immunostaining. The stained cells were counted from 10 different randomly selected fields per slice. As shown in Fig. 5c, proliferative cells appeared brown and there were no visible differences in the number of Ki-67^+^ cells between the control and experimental groups (Fig. 6e). These findings implied that RF exposure had no obvious effect on the development or progression of glial tumors.

### Gene and protein expression

**Fig. 7:**
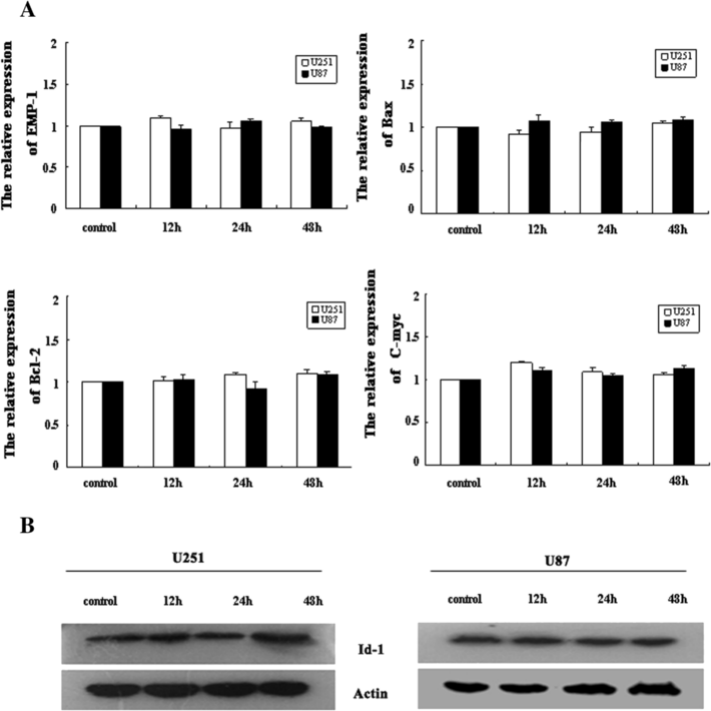
Gene and protein expression levels in human glioma cells after exposure. **a** Real-time PCR analysis of *bcl-2*, *bax*, *EMP-1*, and *c-myc* expression in sham- or RF-exposed cells. The average of the normalized ratio of the target gene compared with actin was calculated. The relative expression levels of genes were expressed as a value of treated cells in different groups compared with a value of untreated cells. Data represent means ± SD from three independent experiments. **b** Analysis of Id-1 protein expression levels in U251-MG and U87-MG cells by western blotting. β-actin was used as a loading control for standardization

The development of tumors can be regulated by a series of genes, including proliferative genes (*c-myc*, *emp-1*) and apoptotic genes (*bax*, *bcl-2*). Therefore, the possible contribution of these genes in response to RF exposure in U87-MG and U251-MG cells was examined by quantitative RT-PCR. Our findings showed that EMF exposure to human glioblastoma cell lines did not significantly affect transcript levels of these genes (Fig. 7a).

Furthermore, western blot analysis was performed in whole cell extracts from U87-MG and U251-MG cells that were either unexposed or exposed to RF for 12, 24, and 48 h. There were no differences in gene or protein expression between the control and experimental groups following 12, 24, or 48 h exposure (Fig. 7b).

## Discussion

Radiofrequency electromagnetic field in the human environment is mainly caused by mobile communication systems. The high levels of anthropogenic EMF in the contemporary environment raise questions about their effects on human health. Epidemiological studies have been performed to elucidate the potential hazardous effects of EMF from electrical wiring and equipment. Three meta-analyses of case-control studies concluded that using cell phones for more than 10 years was associated with an increase in the overall risk of developing a brain tumor. However, The Interphone Study [12], the largest health-related case-control international study of the use of cell phones and head and neck tumors, showed no statistically significant increases in brain cancers related to higher amounts of cell phone use. These studies highlight the debate about the involvement of mobile telephones in the development and/or progression of neurological disorders and cancers [21–25].

Since the development of 3G technologies, which replaced GSM and CDMA systems, TD-SCDMA has become widely used in China. However, to date, there are only a few reports about the effects of TD-SCDMA on the development of human brain cancers. Additionally, most patients want to know whether the use of mobile telephones affects their health or a pre-existing illness. Therefore, this study aimed to investigate whether 1950-MHz TD-SCDMA exposure could affect the biological features of human brain tumor cell lines and promote their growth *in vivo*.

Alterations in growth rate are one of the most sensitive parameters that can be used to elucidate the cellular stress response to environmental carcinogens. Cell growth depends upon the balance between cell proliferation and apoptosis. There are many reports about the effects of EMF on cell proliferation and apoptosis; however, they have yielded conflicting results. Merola *et al.* reported that exposure to a radiofrequency of 900 MHz for up to 72 h does not induce significant alterations in either proliferation or apoptosis in glioblastoma cell lines [26]. By contrast, Caraglia *et al.* exposed human oropharyngeal epidermal carcinoma KB cells to a 1.95-GHz RF signal for 1, 2, or 3 h and observed a time-dependent increase in the levels of apoptosis, along with a time-dependent reduction in heat shock protein levels [27]. We thought that different types of cells and exposure systems might be responsible for these conflicting data. Herein, we screened different glioblastoma- and neuroblastoma-derived cell lines. Human glioblastomas are the most frequent types of human brain tumors and are a major cause of mortality. Thus, several glioblastoma cell lines were selected. The CCK-8 and Annexin V/PI assays were performed to evaluate the sensitivity of different human glioblastoma cell lines (T98G, A127, U251-MG, and U87-MG) to 1950-MHz TD-SCDMA EMF. Our data confirmed the failure of 1950-MHz TD-SCDMA exposure to induce significant modifications in growth rates or the apoptosis ratio of all human glioblastoma cells after exposure for 48 h. We speculate that RF-induced changes in cell proliferation and apoptosis might depend either on the duration of exposure or the lag-time between RF exposure and the completion of the cell population-doubling time. T98G is hyperpentaploid. Additionally, neither T98G nor A127 cells could form tumors in nude mice. Thus, U87-MG and U251-MG, which represent the most frequent types of human brain tumors [19], were used for more detailed subsequent studies.

The ability of 1950-MHz TD-SCDMA RF fields to affect the cell cycle of U87-MG and U251-MG cells was examined. Cells exposed for 12, 24, or 48 h (after exposure for 12 or 24 h, cells were cultured in an RF-free environment up to 48 h post-exposure) were subjected to cell cycle assays. No substantial effects on the cell cycle were identified between sham and exposed cells cycle after exposure to 1950-MHz TD-S-CDMA for 12–48 h. Interestedly, the cell phase distribution of U87-MG cells differed from that of U251-MG cells. We speculate that the different growth rates of these two cell lines might contribute to the different cell phase distributions. These results were in agreement with the growth and apoptosis assays and implied that 1950-MHz TD-SCDMA RF exposure for up to 48 h might have no obvious effects on human glioma cells.

Trans-well and wound healing assays were used to assess the influence of RF on tumor promotion *in vitro*. We found that 1950-MHz TD-SCDMA RF did not alter the invasion or migration ability of U87-MG or U251-MG cells. Furthermore, *in vivo* xenograft experiments also indicated that 1950-MHz TD-SCDMA RFs did not alter the features of human glioblastoma cells. Therefore, 1950-MHz TD-SCDMA RF fields might not act as a tumor-promoting agent.

Additionally, we investigated gene and protein expression in U87-MG and U251-MG cells in response to mobile phone exposure. Apoptosis or tumor-related genes including, *c-myc*, *Emp-1*, *bax*, and *bcl-2*, play important roles in cell cycle progression, apoptosis, and cellular transformation. Mutations in these genes have been associated with tumor development and progression [28–30]. Id-1 is involved in cell growth, development, maturity, and death control processes. Its expression can promote cell proliferation and inhibit differentiation. Moreover, the expression of Id-1 in tumor cells has been associated with tumor malignancies [31]. Thus, to elucidate the effects of RF exposure on growth, apoptosis, and malignancy of human glioma cells, we analyzed *c-myc*, *Emp-1*, *bcl-2*, and *bax* expression by real-time PCR and Id-1 protein levels by western blotting. No significant changes in gene or protein expression profiles were observed for any RF signal tested in either U251-MG or U87-MG cells.

Finally, to test whether these cells were particularly resistant to the stimuli that we administered, U251-MG and U87-MG cells were treated with staurosporine. The changes in the apoptosis profile of U251-MG and U87-MG exposed to staurosporine were significantly different from those of these cells exposed to 1950-MHz TD-SCDMA EMF (Additional file 4: Figure S2). Significant apoptosis of U251-MG and U87-MG cells was accompanied by significantly increased expression of *bax* and reduced levels of *bcl-2* in the staurosporine-treated cells (Additional file 4: Figure S2). These findings indicated that our system could detect differences in the cells activated by certain stimuli. This is the first study to systemically explore the effects of 1950-MHz TD-SCDMAs, which are one of the 3G technologies now widely used in China, on two types of human glioma cells. These results are of great importance to properly understand the relationship between the use of 3G mobile telephones and the risk of brain tumor development.

## Conclusion

Previously, we found that 1950-MH TD-SCDMA EMF did not affect the growth of rat glioma cells at 12 or 48 h. In this present study, we further examined the growth, cell cycle, apoptosis, migration, invasion, and malignancy of human glioma cells after exposure to the same conditions and confirmed that 1950-MH TD-SCDMA EMF did not act as a tumor promoter.

## Additional files

Additional file 1: Figure S3. The method of SAR calculation. (A) Simulation model demonstration. The D1950V3 dipole is a 1/4 wavelength Balun dipole antenna, and rectangle cell culture container is used to contain cell culture and cells. The antenna is used as exposure source and cell culture in the container is exposed. The shell of the container is made of low loss material, with the thickness of 2 mm. The dipole dimension and characters used is same as its manufacture’s handbook by Schmid & Partner Engineering AG (SPEAG), the Swiss Federal Polytechnic University, Switzerland. The distance between antenna and the container is 10 mm. In actual experiment, the distance is assured by 10 mm spacer, which is accompanied with the antenna by SPEAG. The simulation area dimension is 50 mm × 50 mm × 50 mm and the grid step is 5 mm, which is complied with IEC 62209-1.(B) SAR calculation data. The blue curve is peak SAR, and the red curve is 10 g averaged SAR. The distance between antenna and the container is 10 mm, and the container shell thickness is 2 mm. Additional file 2: Figure S1. Effects of RF emission on different glioblastoma cell lines. (A) The proliferation of A172 and T98G cells was measured using the CKK-8 assay from days 1 to 6 after exposure. Data represent means ± SD from five independent experiments. (B) Apoptosis of A172 and T98G cells were detected using the FITC-conjugated Annexin-V/PI assay after exposure for 48 h. (C) Statistical analysis of the percentage of apoptotic cells in the different groups. Data represent means ± SD from three independent experiments. Additional file 3: Table S1. The ratio of apoptosis in different groups. **Table S2.** The distribution of cell cycle in different groups. Additional file 4: Figure S2. Effects of staurosporine on glioblastoma cell lines. (A) Apoptosis of U87-MG and U251-MG cells were detected using the FITC-conjugated Annexin-V/PI assay after staurosporine treatment for 24 h. (B) Real-time PCR analysis of *bcl-2, bax* expression in sham- or staurosporine- treated cells. The average of the normalized ratio of the target gene compared with actin was calculated. The relative expression levels of genes were expressed as a value of treated cells in different groups compared with a value of untreated cells. Data represent means ± SD from three independent experiments. *comparing with Control value < 0.05.

## Abbreviations

TD-SCDMA: Time division-synchronous code division multiple access
RF: Radiofrequency
