# Supplementary figures and images for: Exposure to 3G mobile phone signals does not affect the biological features of brain tumor cells
Source: BMC Public Health. 2015 Aug 8;15:764. doi: 10.1186/s12889-015-1996-7 (PMC4529714; doi:10.1186/s12889-015-1996-7)

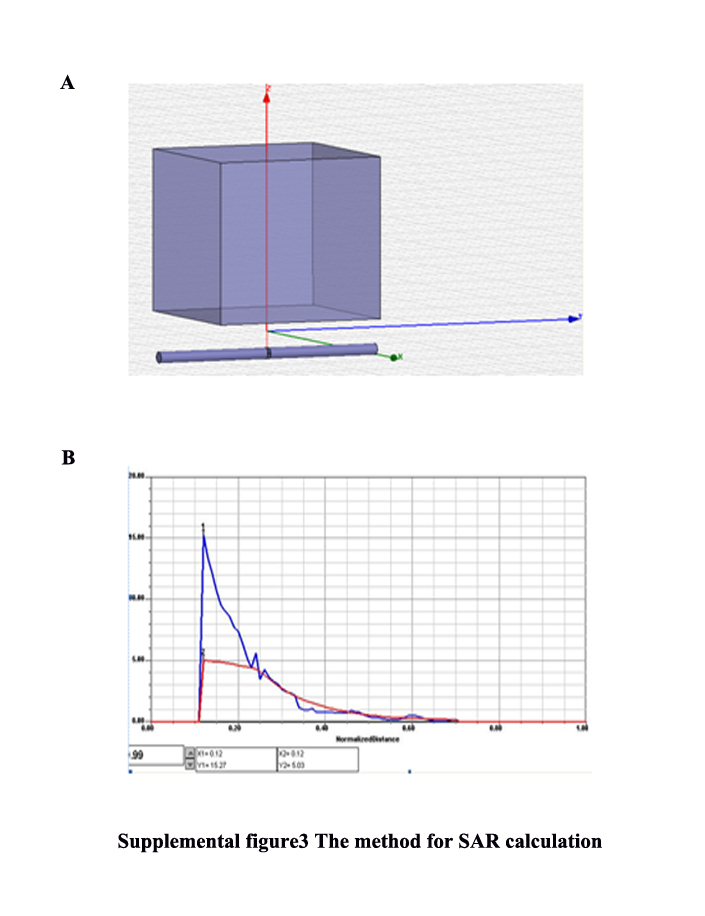

Supplement: Additional file 1: Figure S3. — The method of SAR calculation. (A) Simulation model demonstration. The D1950V3 dipole is a 1/4 wavelength Balun dipole antenna, and rectangle cell culture container is used to contain cell culture and cells. The antenna is used as exposure source and cell culture in the container is exposed. The shell of the container is made of low loss material, with the thickness of 2 mm. The dipole dimension and characters used is same as its manufacture’s handbook by Schmid & Partner Engineering AG (SPEAG), the Swiss Federal Polytechnic University, Switzerland. The distance between antenna and the container is 10 mm. In actual experiment, the distance is assured by 10 mm spacer, which is accompanied with the antenna by SPEAG. The simulation area dimension is 50 mm × 50 mm × 50 mm and the grid step is 5 mm, which is complied with IEC 62209-1.(B) SAR calculation data. The blue curve is peak SAR, and the red curve is 10 g averaged SAR. The distance between antenna and the container is 10 mm, and the container shell thickness is 2 mm. [file 12889_2015_1996_MOESM1_ESM.jpeg]

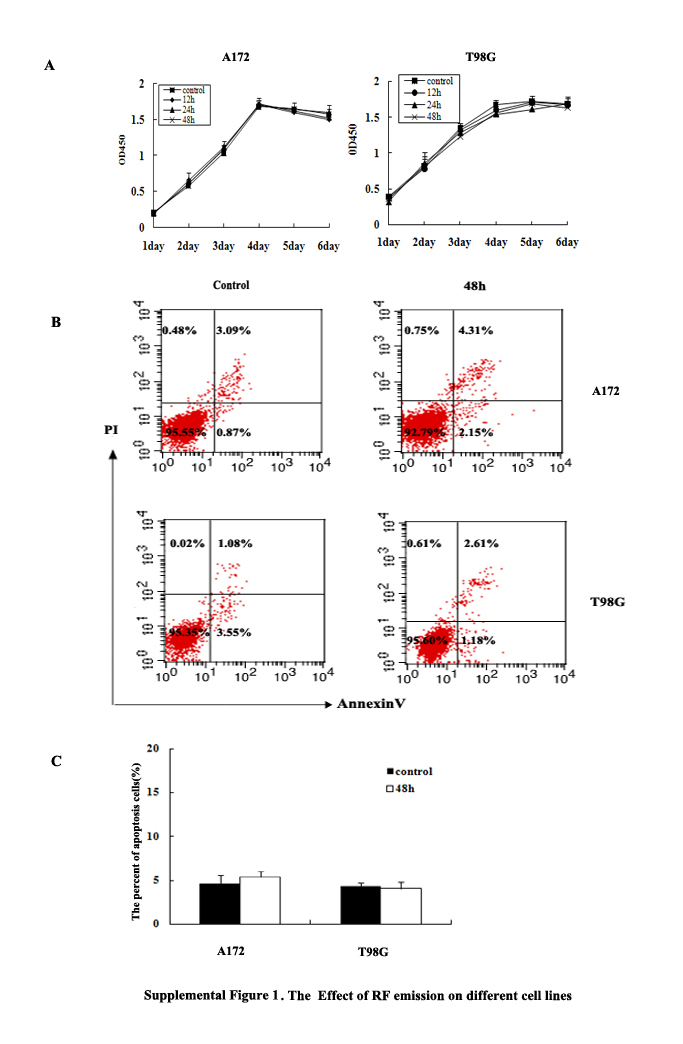

Supplement: Additional file 2: Figure S1. — Effects of RF emission on different glioblastoma cell lines. (A) The proliferation of A172 and T98G cells was measured using the CKK-8 assay from days 1 to 6 after exposure. Data represent means ± SD from five independent experiments. (B) Apoptosis of A172 and T98G cells were detected using the FITC-conjugated Annexin-V/PI assay after exposure for 48 h. (C) Statistical analysis of the percentage of apoptotic cells in the different groups. Data represent means ± SD from three independent experiments. [file 12889_2015_1996_MOESM2_ESM.jpeg]

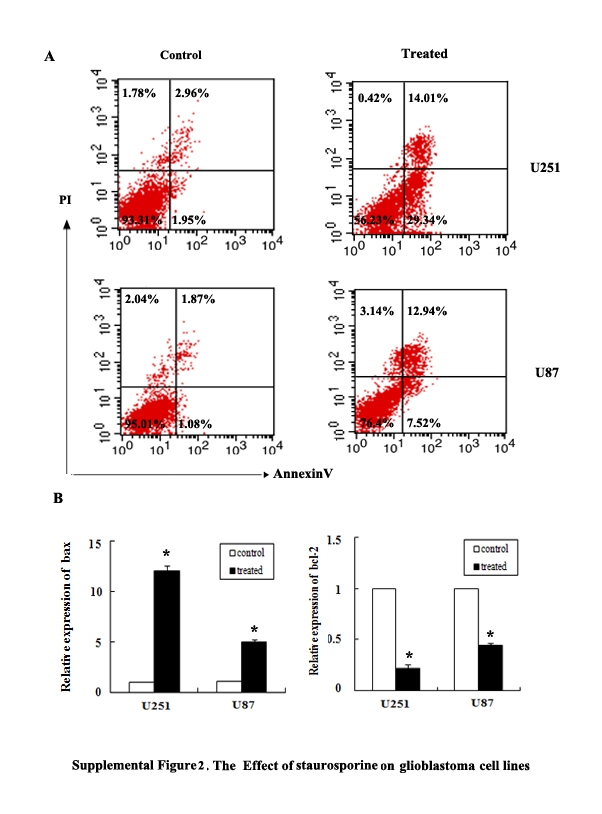

Supplement: Additional file 4: Figure S2. — Effects of staurosporine on glioblastoma cell lines. (A) Apoptosis of U87-MG and U251-MG cells were detected using the FITC-conjugated Annexin-V/PI assay after staurosporine treatment for 24 h. (B) Real-time PCR analysis of bcl-2, bax expression in sham- or staurosporine- treated cells. The average of the normalized ratio of the target gene compared with actin was calculated. The relative expression levels of genes were expressed as a value of treated cells in different groups compared with a value of untreated cells. Data represent means ± SD from three independent experiments. *comparing with Control value < 0.05. [file 12889_2015_1996_MOESM4_ESM.jpeg]
