# Supplementary material for: Exposure to 3G mobile phone signals does not affect the biological features of brain tumor cells
Source: BMC Public Health. 2015 Aug 8;15:764. doi: 10.1186/s12889-015-1996-7 (PMC4529714; doi:10.1186/s12889-015-1996-7)
Supplement: Additional file 3: Table S1. — The ratio of apoptosis in different groups. Table S2. The distribution of cell cycle in different groups. [file 12889_2015_1996_MOESM3_ESM.doc]

Supply table 1. The ratio of apoptosis in different groups

|  | Control | 12h | 24h | 48h |
| --- | --- | --- | --- | --- |
| U251 | 5.2%±0.67% | 5.0%±0.9% | 4.7%±0.66% | 5.6%±0.34% |
| U87 | 5.5%±0.8% | 6.1%±1.8% | 5.8%±0.7% | 6.4%±1.7% |

Supply table 2. The distribution of cell cycle in different groups

|  |  | Control | 12h | 24h | 48h |
| --- | --- | --- | --- | --- | --- |
| U251 | G0-G1 | 85.4%±2.5% | 86.3%±2.9% | 85.5%±3.2% | 84.1%±2.3% |
|  | G2-M | 7.4%±1.2% | 7.0%±1.4% | 7.3%±1.5% | 7.7%±1.8% |
|  | S | 7.2%±1.6% | 6.6%±2.5% | 7.1%±2.8% | 8.2%±1.1% |
| U87 | G0-G1 | 54.51%±2.4% | 53.09%±5.3% | 53.85%±2.7% | 55.01%±2.5% |
|  | G2-M | 3.92%±0.41% | 4.27%±0.28% | 4.25%±0.95% | 4.62%±0.5% |
|  | S | 41.67%±2.7% | 42.64%±5.1% | 41.9%±2.1% | 40.37%±1.1% |
